# Arg-type dihydroflavonol 4-reductase genes from the fern *Dryopteris erythrosora* play important roles in the biosynthesis of anthocyanins

**DOI:** 10.1371/journal.pone.0232090

**Published:** 2020-05-01

**Authors:** Xuefei Chen, Wenli Liu, Xianyan Huang, Huanhuan Fu, Quanxi Wang, Youfang Wang, Jianguo Cao

**Affiliations:** 1 College of Life Science, East China Normal University, Shanghai, China; 2 College of Life Science, Shanghai Normal University, Shanghai, China; 3 Shanghai Key Laboratory of Plant Functional Genomics and Resource, Shanghai Chenshan Botanical Garden, Shanghai, China; Northeast Forestry University, CHINA

## Abstract

Dihydroflavonol 4-reductase (DFR), a key enzyme involved in the biosynthesis of anthocyanins, has been cloned from various species. However, little research has been conducted on this enzyme in ferns, which occupy a unique evolutionary position. In this study, we isolated two novel DFR genes from the fern *Dryopteris erythrosora*. *In vitro* enzymatic analysis revealed that DeDFR1 and DeDFR2 enzymes can catalyze dihydrokaempferol and dihydroquercetin but cannot catalyze dihydromyricetin. Amino acid sequence analysis showed that DeDFR1 and DeDFR2 have an arginine at the same substrate-specificity-determining site as that in the ferns *Salvinia cucullata* and *Azolla filiculoides*. Thus, we speculate that the Arg-type DFR is a new DFR functional type. To further verify the substrate preferences of the Arg-type DFR, an amino acid substitution assay was conducted. When N133 was mutated to R133, Arabidopsis DFR protein completely lost its catalytic activity for dihydromyricetin, as observed for DeDFR1 and DeDFR2. Additionally, heterologous expression of *DeDFR2* in the Arabidopsis *tt3-1* mutant resulted in increasing anthocyanin accumulation. In summary, *DeDFR1* and *DeDFR2* are considered to be a new type of DFR with unique structures and functions. The discovery of the Arg-type DFR provides new insights into the anthocyanin biosynthesis pathway in ferns.

## Introduction

Anthocyanins represent an important subgroup of flavonoids, which are important secondary metabolites produced by plants to adapt to the terrestrial environment. Anthocyanins, serving as pigments, signaling molecules, photoprotectants, and protectants against biotic and abiotic stress, have a broad spectrum of functions in plant development [1–3]. Anthocyanins in food plants are potentially valuable components of the human diet. In addition, they have antioxidant, anticancer, and anti-inflammatory activities and may contribute to the prevention and treatment of metabolic disorders, cancer, eye diseases and cardiovascular diseases [4–6].

Dihydroflavonol 4-reductase (DFR; EC1.1.1.219) is a pivotal enzyme in anthocyanin biosynthesis [7]. DFR can use NADPH as a cofactor to reduce dihydroflavonols, including dihydrokaempferol (DHK), dihydroquercetin (DHQ), and dihydromyricetin (DHM), to the corresponding flavan-3,4-diols (leucoanthocyanidins), which are the precursors of anthocyanidins and proanthocyanidins [8–11]. *DFR* genes have been cloned from various species, such as *Arabidopsis thaliana*, *Brassica rapa*, *Ginkgo biloba*, *Lotus japonicus* and *Zea mays* [12–17]. The DFR mutation (*tt3*) in *A*. *thaliana* has been demonstrated to cause anthocyanin loss, and the color of the seed coat changes to yellow [18–20]. Targeted mutagenesis of the *InDFR-B* gene by CRISPR/Cas9 technology leads to the production of anthocyanin-free white flowers in *Petunia* [21]. In a study of transgenic sweet potato, inhibition of *IbDFR* expression significantly reduces anthocyanin accumulation and antioxidant capacity [22]. In addition, overexpression of DFR can increase anthocyanin production in tobacco and apple [23, 24]. DFRs not only regulate the total anthocyanin content but also have substrate preferences that affect the accumulation of different types of anthocyanins. Crystal structure analysis of the *Vitis vinifera* DFR shows that this protein belongs to the short-chain dehydrogenase/reductase family, and the 131–156 region is the substrate-binding site [25]. Some studies have indicated that amino acid residue 133 plays an important role in substrate specificity and classified DFRs into different types based on this site. DFRs in most plants are Asn-type DFRs, which can catalyze three types of substrates (DHK, DHQ, DHM). Some species, such as *Petunia* and *Cymbidium* species, have Asp-type DFRs, which do not have the ability to catalyze DHK. Therefore, these species do not produce brick red/orange flowers with pelargonidin-based anthocyanins [26, 27]. Thus far, most genes have been isolated from seed plants, and few studies on the function of *DFR* genes in spore plants.

Ferns are members of vascular plants that reproduce via spores and have neither seeds nor flowers. Therefore, ferns have a unique evolutionary position between bryophytes and seed plants. *Dryopteris erythrosora* O. Kuntze is a common garden ornamental fern with young fronds that appear red to bronze throughout the spring. Recent studies have revealed that *D*. *erythrosora* is a flavonoid-rich plant with a total flavonoid content of more than 7%, containing at least 8 flavonols, 4 flavones, 3 chalcones, 2 flavanols, 2 flavanones, 1 homoisoflavone, 1 isoflavone, and 1 isoflavanone [28, 29]. Additionally, the flavonoid extract of *D*. *erythrosora* has antioxidant, anticancer and acetylcholinesterase inhibitor activities [30]. In addition, *D*. *erythrosora* was one of the first ferns to be reported to contain anthocyanins, which mainly contain cyanidin and delphinidin [31]. However, only a few studies have examined anthocyanin biosynthesis in *D*. *erythrosora*, and the pathways in anthocyanin biosynthesis of ferns are still unclear. Previous studies have identified two upstream genes in *D*. *fragrans*, namely, 4-coumarate:coenzyme A ligase (4CL) and chalcone synthase (CHS) [32, 33]. Campanella, et al. analyzed the phylogenetic relationship of the DFR gene from the lycophyte *Selaginella moellendorffii*, fern *Adiantum capillus-veneris* and other plants, but the function of DFR enzymes in ferns have not been characterized [34]. In this paper, by analyzing transcriptome data, we isolated two new *DFRs* from *D*. *erythrosora*, designated *DeDFR1* and *DeDFR2*. *DeDFRs* have unique residues in the region that determines substrate specificity, and these two DFRs can catalyze DHK and DHQ *in vitro*. Using site-directed mutagenesis, we analyzed the relationship between the region that determines substrate specificity and the substrate preferences of DFRs. In addition, we investigated the potential roles of *DeDFRs* in plants by introducing *DeDFRs* into Arabidopsis *DFR* (*tt3-1*) mutant plants. The results suggest that *DeDFR1* and *DeDFR2* are associated with color development and are helpful for understanding anthocyanin biosynthesis in ferns.

## Materials and methods

### Plant materials

*Dryopteris erythrosora* was grown in a field or in pots in a growth chamber at the College of Life Science, Shanghai Normal University, China. For gene expression analysis, we sampled rolled immature fronds, young fronds and mature fronds from plants grown in the field. All fronds were frozen in liquid nitrogen and stored at -80°C. The *Arabidopsis thaliana* ecotype Columbia (Col) was a gift from Prof. Zhongnan Yang (Shanghai Normal University). The Arabidopsis *tt3-1* mutant was purchased from SALK (SALK ID: CS2114).

### Isolation and cloning of *DeDFR1* and *DeDFR2*

Total RNA was extracted from 0.5 g of rolled immature fronds of *D*. *erythrosora* using the RNApure Plant Kit (Cwbiotech, China). First-strand cDNA was subsequently synthesized using the Superscript III First-Strand Synthesis Kit (Invitrogen, USA). All steps were performed by following the manufacturer’s instructions supplied with the kits. The transcriptome was sequenced on the Illumina HiSeq2000 platform (Illumina, USA) at BGI (Shenzhen, China).

Based on the assembled transcriptomic sequences of rolled immature fronds of *D*. *erythrosora* (TSA number: GHEW00000000), we designed pairs of specific primers (S1 Table) for DeDFR1 and DeDFR2. TaKaRa LA Taq (Takara, Japan) was used for PCR. The thermal cycling conditions were as follows: 94°C for 1 min, followed by 30 cycles of 94°C for 30 s, 55°C for 30 s, and 72°C for 60 s, and a final extension step at 72°C for 5 min. The PCR products were ligated to the pEASY-T5 Zero vector (Transgen Biology, China), and single colonies were selected for sequencing.

### Phylogenetic analysis

Multisequence alignment of amino acid sequences of DFRs from other plants and deduced amino acid sequences of *DeDFR1* and *DeDFR2* was performed using DNAMAN. The phylogenetic tree was constructed using the neighbor-joining method by using MEGA 5.0 software. The GenBank accession numbers for the DFR sequences from these plants are as follows: *A*. *thaliana* (AED94866.1), *Bromheadia finlaysoniana* (AAB62873.1), *Callistephus chinensis* (CAA91922.1), *Daucus carota* (AAD56578.1), *Ginkgo biloba* (AGR34043.1), *Ipomoea purpurea* (BAA74700.1), *Iris x hollandica* (BAF93896.1), *Lilium hybrid* (BAB40789.1), *Lotus japonicus1* (BAE19948.1), *L*. *japonicus2* (AAV71171.1), *L*. *japonicus3* (BAE19950.1), *L*. *japonicus4* (BAE19951.1), *L*. *japonicus5* (BAE19953.1), *Medicago truncatula1* (XP_013466134.1), *M*. *truncatula2* (XP_013466133.1), *Nicotiana tabacum* (BAF96936.1), *Oryza sativa* (BAA36183.1), *Petunia hybrid* (AGI96402.1), *Phoenix dactylifera* (XP_008797532.1), *Selaginella moellendorffii1* (XP_002984902.1), *S*. *moellendorffii2* (XP_002985959.1), *Vitis vinifera* (NP_001268144.1), and *Zea mays* (NP_001152467.2). The DFR sequences from *Azolla filiculoides* and *Salvinia cucullata* were found from the CoGe (https://genomevolution.org/coge/), and the GenBank accession numbers are *A*. *filiculoides*1-6 (MN939152-MN939157) and *S*. *cucullata*1-5 (MN939158-MN939162).

### Expression analysis of DeDFR1 and DeDFR2

The expression levels of *DeDFR1* and *DeDFR2* in *D*. *erythrosora* were analyzed using qRT-PCR. The cDNA used for detection of expression was synthesized by using PrimeScript^™^ RT Master Mix (Takara). The PCR primers were designed using Primer Premier 5.0 and are listed in S1 Table. All reactions were carried out with an ABI 7500 real-time PCR system (Applied Biosystems, USA) with a 20-μL reaction system consisting of 10 μL of SYBR Green^™^ Premix Ex Taq^™^ (Takara), 0.4 μL of ROX Reference Dye II, 0.2 μM each specific primer, and 10 ng of template cDNA. The thermal cycling conditions were as follows: an initial step of 95°C for 30 s, followed by 40 cycles of 95°C for 5 s and 60°C for 34 s. A melting curve was generated for each sample at the end of each run to verify the purity of the amplified products, and at least three replicates of each sample were analyzed. The transcript levels were normalized using *DeGAPDH* (GenBank: MK920229) as the internal control and calculated using the 2^(−ΔΔCt)^ analysis method.

### Determination of anthocyanidin content

Fern leaf (0.2 g dry weight) extracts were prepared with 4 mL of extraction solution (ethanol:water:hydrochloric acid = 2:1:1), ultrasonic extraction for 30 min, and hydrolysis in boiling water for 1 h. The supernatant was then filtered through a 0.22-μm reinforced nylon membrane filter (Millipore, USA). Samples were detected using a Shimadzu LC-20AD instrument equipped with an SPD-M20A photodiode array detector (Shimadzu, Japan). Twenty microliters of the extract was separated with a Symmetry column (C18, 250 × 4.6 mm, 5 μm, Waters, USA) and detected at 530 nm. The column oven temperature was set at 35°C. The mobile phase consisted of 1% formic acid (A) and acetonitrile (B) with the following gradient program: 0–2 min, 8–12% B; 2–5 min, 12–18% B; 5–10 min, 18–20% B; 10–12 min, 20–25% B; 12–15 min, 25–30% B; 15–18 min, 30–45% B; 18–20 min, 45–80% B; 20–22 min, 80–8% B; 22–30 min, 8% B. The flow rate was 0.8 mL/min. Cyanidin chloride, delphinidin chloride and pelargonidin chloride (Sigma-Aldrich, USA) were used as standards. The process was repeated at least three times for each sample.

### Production of recombinant DFR proteins in *Escherichia coli*

*DeDFR1* and *DeDFR2* were cloned into the expression vector pET28a (+) (Novagen, USA) by using the In-Fusion PCR Cloning Kit (Takara). The PCR primers were designed using In-Fusion Cloning tools (https://www.takarabio.com). *Bam HI* and *Sac I* were used as the two restriction sites. The list of primers is provided in S1 Table. In-Fusion cloning was performed according to the manufacturer’s instructions at 50°C for 15 min with a 5-μL reaction system consisting of 1 μL of 5× In-Fusion HD Enzyme Premix, 50 ng of linearized vector and 25 ng of purified PCR fragment. Ligated products were introduced into competent *Trans5α* cells (Transgen Biology) for sequencing. After confirmation of the sequences, the subcloned vectors were used to transform *E*. *coli* strain BL21 (DE3) (Transgen Biology). The empty vector and pET28a-AtDFR were also introduced into *E*. *coli* as controls.

The expression of each recombinant DFR protein was induced by 0.2 mM isopropyl-thio-β-D-galactoside (IPTG) in LB culture for 20 h at 20°C. The *E*. *coli* cells were harvested by centrifugation and resuspended in extraction buffer (25 mM Tris-HCl, pH 8.0). The cells were lysed by sonication, and the debris was removed by centrifugation at 8,000 rpm for 5 min at 4°C. The protein concentration was estimated by using the Easy Protein Quantitative Kit (Transgen Biology), and the protein quality was examined using 10% SDS-PAGE.

### Enzyme assay

Measurements of the activity of the recombinant DeDFR1 and DeDFR2 were performed according to the methods of Petit et al. and Katsu et al. with minor modifications [25, 35]. A 500-μL reaction mixture consisted of 1.6 mM NADPH, 50 mM Tris-HCl (pH 7.0), 0.8 mM substrate, and 5 mg of total protein. Three dihydroflavonols were selected as experimental substrates: dihydrokaempferol (DHK), dihydroquercetin (DHQ) and dihydromyricetin (DHM) (Sigma-Aldrich). Each reaction was terminated after 30 min at 30°C by extraction with ethyl acetate. Because the obtained leucoanthocyanidins were unstable in solution, the residues were dissolved after evaporation in 200 μL of n-BuOH-HCl (95:5) and incubated at 95°C for 30 min to form anthocyanidins. The HPLC analysis of the DFR products was similar to the anthocyanidin detection described above. The process was repeated at least three times for each sample.

### Amino acid point mutant construction

The 133rd amino acid residue of AtDFR protein (Asn) was modified to Asp or Arg by overlap PCR. The principle of overlap PCR is to design two pairs of primers to amplify sequences with overlapping modified sites and then extend the overlap to obtain the complete sequence. The list of primers is provided in S1 Table. The modified *AtDFR*^*N133D*^ and *AtDFR*^*N133R*^ genes were ligated into the vector pET28a (+), transformed into *E*. *coli* strain BL21 (DE3), and subjected to the above enzymatic assay.

### Plant transformation

To further characterize the function of *DeDFRs*, we cloned *DeDFR1* and *DeDFR2* into pBI121 vector harboring the *CaMV 35S* constitutive promoter by using the In-Fusion PCR Cloning Kit. *Xba I* and *Sma I* were used as the two restriction sites. The list of primers is provided in S1 Table. The recombinant vector was transformed into *Agrobacterium tumefaciens* strain GV3101 using a freeze-thaw method. Seed germination and seeding growth followed the standard Arabidopsis growth protocol. Genetic transformation followed the floral dipping transformation protocol [36]. The growth of infected plants and seed harvesting followed the same protocol as previously reported [9, 37]. T1 seeds were selected on PNS medium with 50 mg L^−1^ kanamycin to obtain T2 plants. Planting of T2 seeds provided more homozygous T3 plants by DNA identification and antibiotic screening. Wild type (Col) and *tt3-1* mutant plants were grown as controls. The Arabidopsis *tubulin* gene was used as an internal control gene to detect the *DeDFRs* expression level.

## Results

### Isolation of *DeDFRs* and phylogenetic analysis

**Fig 1:**
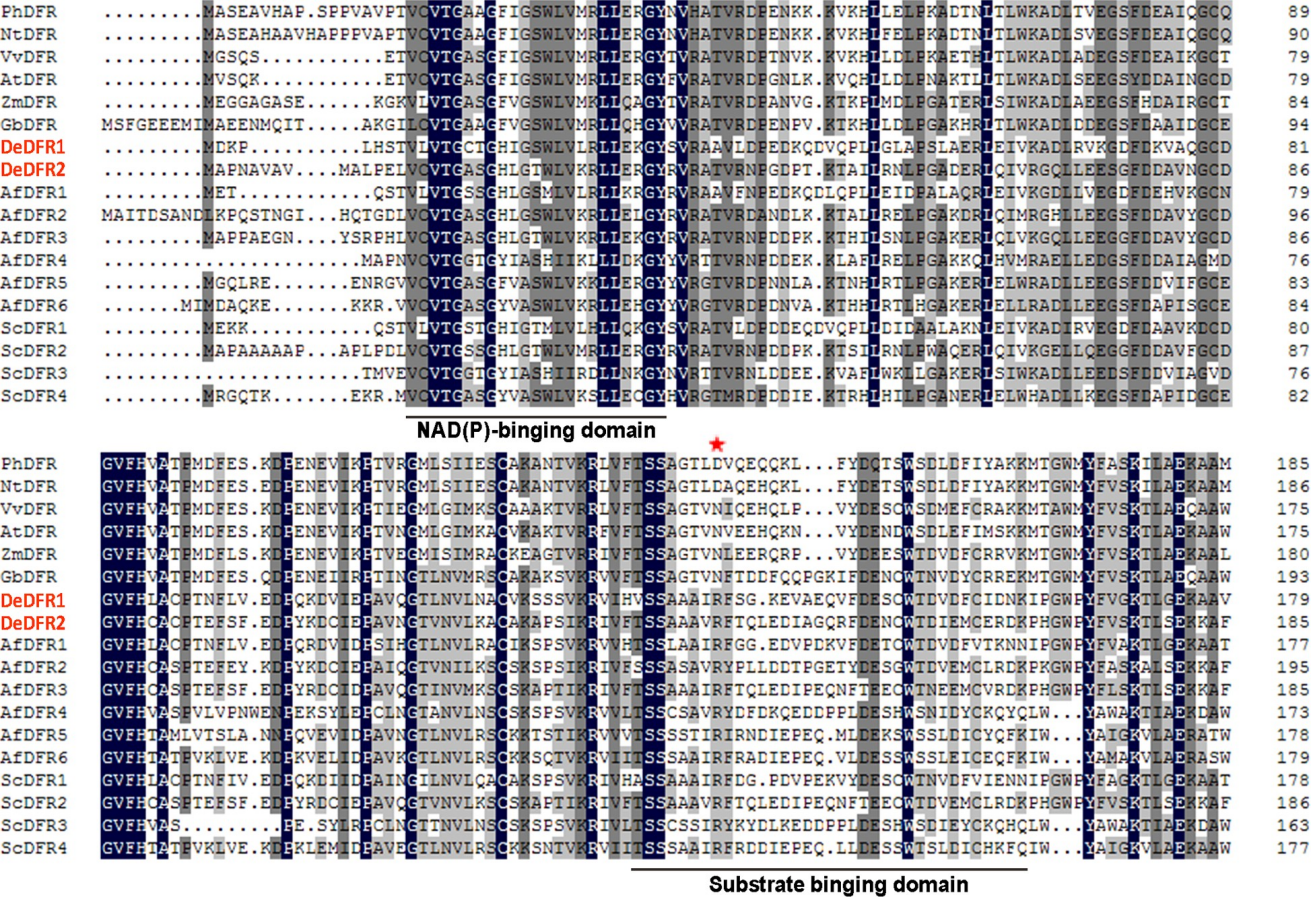
Alignment of the deduced amino acid sequences of DFRs. Identical residues are highlighted in black, and similar residues are highlighted in gray. A putative NAD(P)-binding domain and a putative substrate binding domain are underlined. The red asterisk indicates the 133rd amino acid residue, which is particularly important for substrate specificity. *PhDFR*: *Petunia hybrid*; *NtDFR*: *Nicotiana tabacum*; *VvDFR*: *Vitis vinifera*; *AtDFR*: *Arabidopsis thaliana*; *ZmDFR*: *Zea mays*; *GbDFR*: *Ginkgo biloba*; *DeDFR1-2*: *Dryopteris erythrosora*; *AfDFR1-6*: *Azolla filiculoides*; *ScDFR1-4*: *Salvinia cucullata*.

Based on the assembled transcriptomic sequences of *D*. *erythrosora*, we screened and obtained two *DeDFR* genes (CL10493.Contig3 and CL16087.Contig1) and designated them *DeDFR1* (GenBank: MK920230) and *DeDFR2* (GenBank: MK920231). The open reading frame (ORF) of each *DeDFR* was analyzed, and a pair of specific primers that contained the start and stop codons was designed. The full-length cDNA of each gene was obtained by PCR. The ORF of *DeDFR1* was a 987-bp segment encoding 328 amino acids (Fig 1), and the theoretical molecular weight and isoelectric point were 36.3 kD and 5.41, respectively (calculated using the ExPASy website https://web.expasy.org/compute_pi/, S2 Table). *DeDFR2* was a 1026-bp segment encoding 341 amino acids, and the theoretical molecular weight and isoelectric point were 37.8 kD and 5.54. The identity of the deduced amino acid sequences between two DeDFRs and GbDFR were 45.94% and 47.20%, respectively. These percentages were higher than the identity between the two DeDFRs and AtDFR of 43.48% and 46.34%. In addition, the alignment showed that DeDFR1 and DeDFR2 belonged to the SDR superfamily, including a conserved NAD(P)-binding domain and a substrate-binding domain.

**Fig 2:**
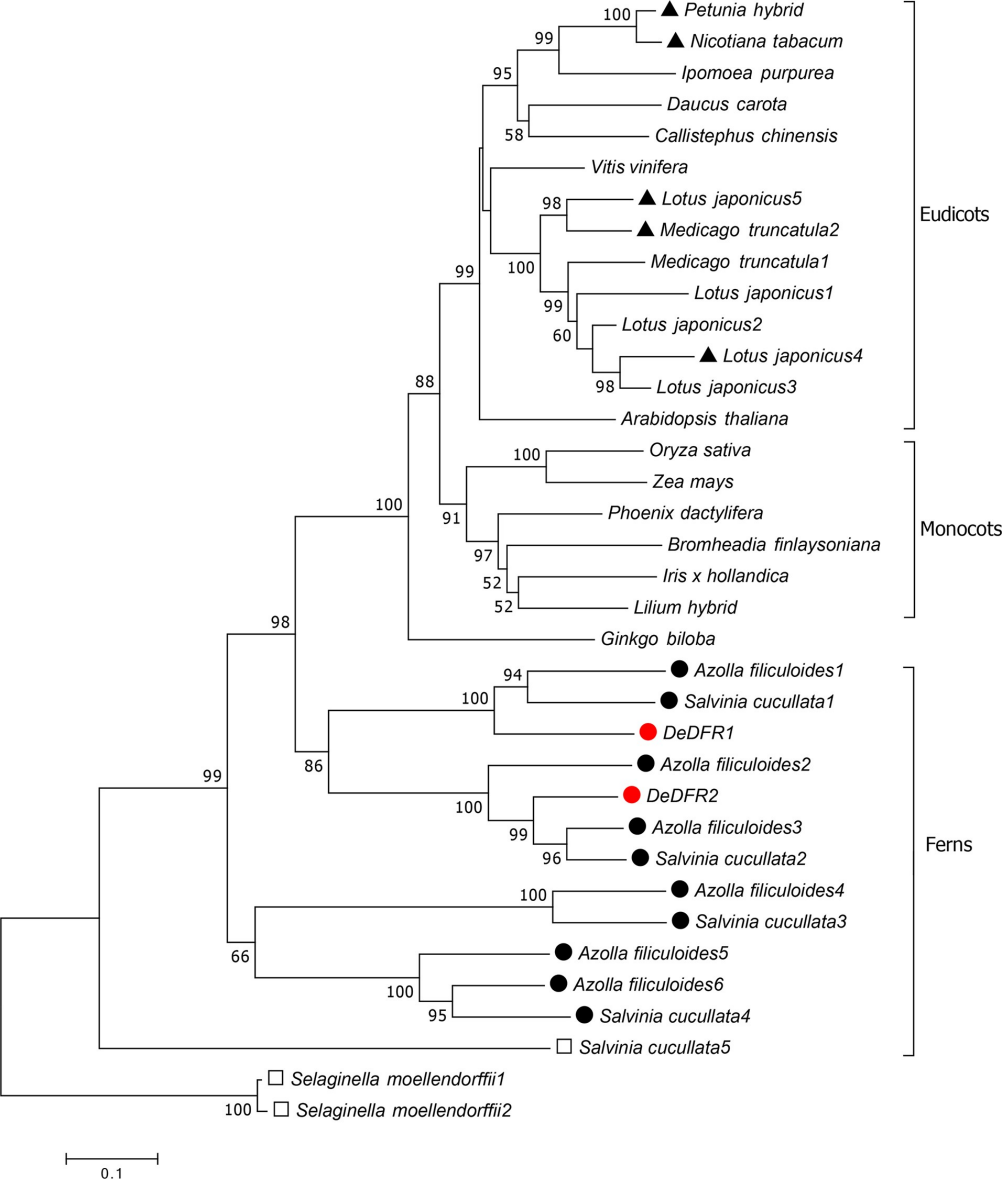
Alignment of the deduced amino acid sequences of DFRs. A phylogenetic tree of DFRs constructed by the neighbor-joining method with 1000 bootstrap replicates. Asn-type (no label), Asp-type (labeled with a filled triangle), Arg-type (labeled with a filled circle) and non-Asn/Asp/Arg-type (labeled with a square) DFRs are labeled with different shapes in front of the species. For accession numbers of the DFR sequences, see Materials and Methods.

Phylogenetic analysis of the deduced amino acid sequences of DeDFR1 and DeDFR2 was performed, and the sequences were compared to those of homologous DFRs from different species (Fig 2). To further investigate the characteristics of DFR proteins in ferns, we found five *Salvinia cucullata DFRs* (*ScDFR1-5*) and six *Azolla filiculoides DFRs* (*AfDFR1-6*) based on genomic data. The DFR sequences of the lycophyte *S*. *moellendorffii* were used as the root of the tree. This phylogenetic tree indicated that DFRs of ferns and seed plants were clearly classified into different branches. Spermatophyte DFRs further diverged into different groups of gymnosperms, monocots, and eudicots. The DFR sequences of ferns were located at the base of the phylogenetic tree. The figure shows that AfDFR1-3, ScDFR1-2 and DeDFR1-2 proteins clustered within a branch, which is more similar to seed plants. It is worth noting that all the DFRs we found from fern *S*. *cucullata*, *A*. *filiculoides* and *D*. *erythrosora* belonged to neither Asn- nor Asp-type DFR. The results indicated that the sequences of DFRs of ferns and seed plants were different; thus, their protein functions may also be different.

### Expression of DeDFRs and anthocyanidin accumulation during frond development

**Fig 3:**
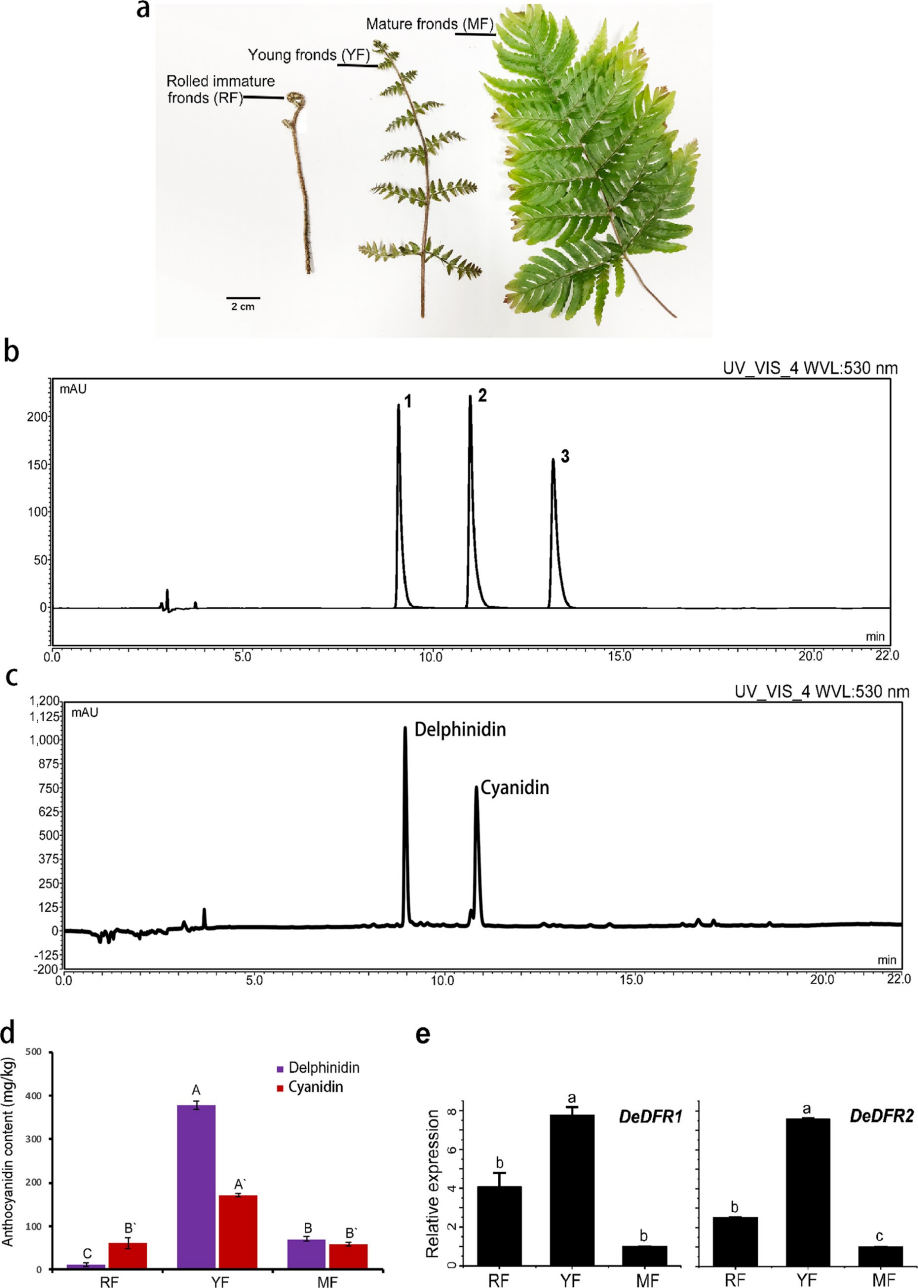
Expression of DeDFRs and anthocyanidin accumulation during frond development in *D*. *erythrosora*. (a) Frond development in *D*. *erythrosora*: rolled immature fronds (RF), young fronds (YF) and mature fronds (MF). (b) HPLC chromatogram of anthocyanidin standards. Peak 1, delphinidin; Peak 2, cyanidin; Peak 3, pelargonidin. (c) The HPLC profile of anthocyanidin levels in young fronds of *D*. *erythrosora* shows the elution times for delphinidin and cyanidin. (d) Accumulation of delphinidin and cyanidin in fronds. Bars represent the mean ± SD. For the delphinidin content, A, B, and C above the bars indicate a significant difference between the samples at *p*<0.01. For the cyanidin content, A' and B' above the bars indicate a significant difference between the samples at *p*<0.01. (e) Relative gene expression levels of *DeDFRs* normalized to the level of *DeGAPDH*. Three replicates were used for each sample. Bars represent the mean ± SD. Different lowercase letters above the bars indicate a significant difference between the samples at *p*<0.05.

In *D*. *erythrosora*, the fronds showed a significant color change during the growth process (Fig 3A). The anthocyanidin component in different stages of frond development was detect by HPLC. At 520 nm, the chromatograms showed that the standard peaks of cyanidin, delphinidin and pelargonidin appeared at approximately 11.09 min, 9.16 min, and 13.22 min, respectively (Fig 3B). The detection results showed that cyanidin and delphinidin, but almost no pelargonidin, were present in these fern fronds (Fig 3C). The contents of both cyanidin and delphinidin were enriched in young fronds (Fig 3D). Cyanidin could be detected in all stages, and delphinidin could hardly be detected in rolled immature fronds. Transcriptomic analysis revealed that *DeDFR2* was present at high levels in raw fragments of the rolled immature fronds, indicating that *DeDFR2* might be closely associated with frond development (Table 1). qRT-PCR was used to examine the expression patterns of *DeDFR1* and *DeDFR2* (Fig 3D). The transcripts of DeDFR1 and DeDFR2 were detected in all fronds tested. The expression of DeDFR1 and DeDFR2 showed an increasing trend at first, followed by a decreasing trend, with frond development. The highest expression was observed in young fronds, followed by rolled immature fronds, and relatively low expression was observed in mature fronds, which is consistent with the change trend observed for cyanidin and delphinidin. Based on the accumulation of anthocyanins and the expression of *DeDFRs*, we speculated that *DeDFR1* and *DeDFR2* might be involved in anthocyanin synthesis.

**Table 1:** The transcripts of *DeDFR1* and *DeDFR2* in rolled immature fronds in *D*. *erythrosora*.

Given name	Unigene Length	ORF Length	Raw fragments	FPKM
DeDFR1	1914	987	1147	13.6767
DeDFR2	1219	1026	25925	485.3722

### Heterologous expression and catalytic activity of DeDFRs

**Fig 4:**
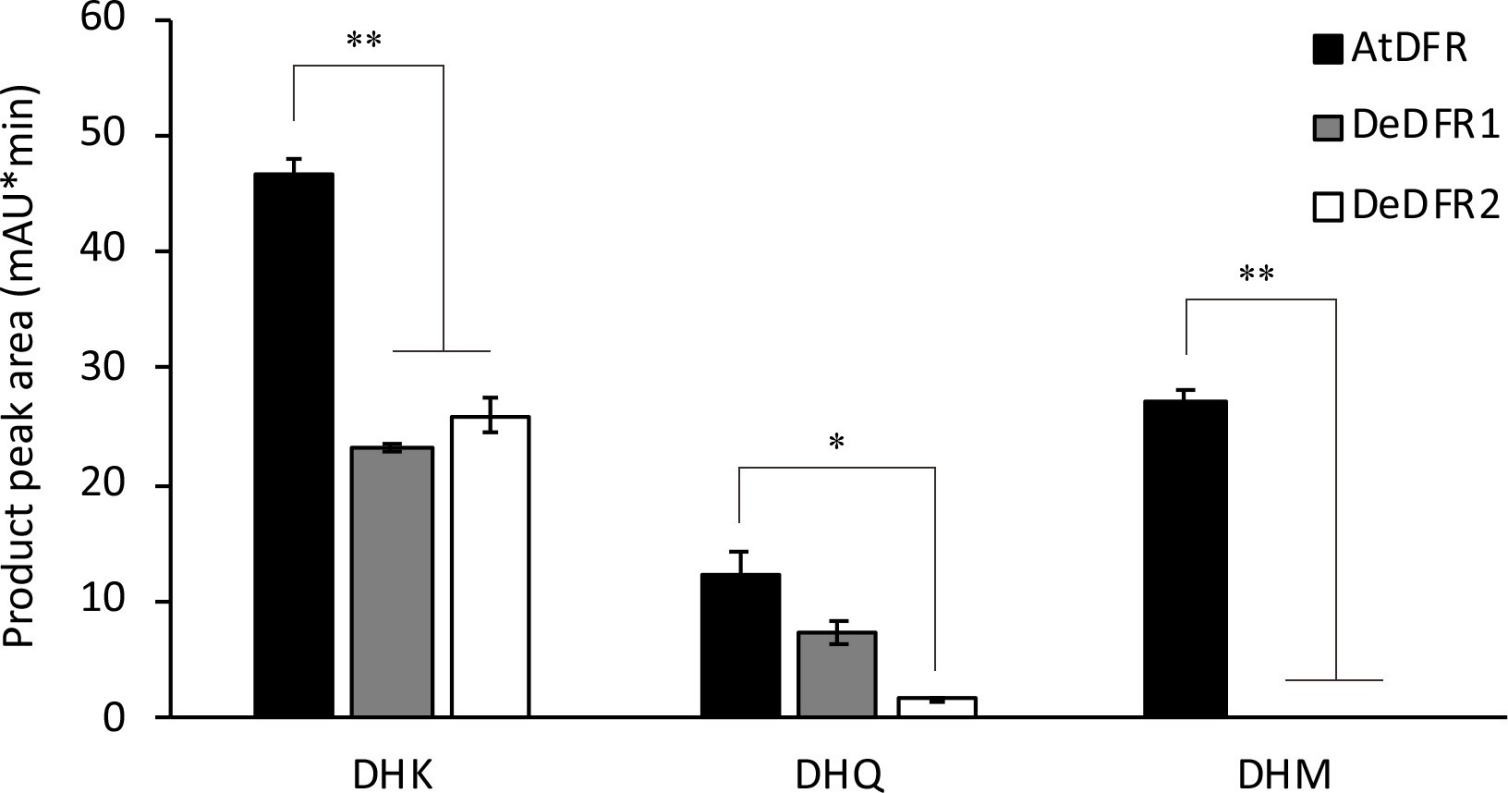
The product peak area of HPLC for DeDFR1, DeDFR2 and AtDFR proteins with DHK, DHQ, and DHM as substrates. Bars represent the mean ± SD. *Statistically significant difference compared with AtDFR at 0.01<*p*<0.05. **Statistically significant difference compared with AtDFR at *p*<0.01.

To identify the catalytic function of DeDFR enzymes on dihydroflavonols, prokaryotic expression was implemented. Using the In-Fusion PCR cloning method, the ORFs of *DeDFR1* and *DeDFR2* were inserted into the expression vector pET28a (+) to obtain recombinant proteins. The SDS-PAGE results showed that these recombinant proteins were similar in total protein content (S1 Fig). The cumbersome purification process tended to inactivate the recombinant protein, and the impure protein obtained after purification affected the accuracy of the protein content determination. Therefore, our activity analysis was based directly on the total protein content. Crude extracts of *Escherichia coli* expressing DFRs were subjected to enzyme assays using DHK, DHQ, and DHM as substrates in the presence of NADPH. The reaction product with an empty vector was colorless, and the HPLC chromatogram showed no significant product peak at 520 nm. Additionally, the results for the positive control AtDFR, an Asn-type DFR, showed that all three dihydroflavonols could be converted to pink anthocyanins, which could be detected by HPLC at 520 nm (S1 Fig). DeDFR1 and DeDFR2 had the ability to catalyze the DHK and DHQ reaction to produce the desired products. However, they could not catalyze DHM to produce the products of AtDFR (S2 Fig). The product peak area in HPLC is shown in Fig 4. The results showed that the catalytic products of DeDFR1 and DeDFR2 were lower than those of AtDFR regardless of the use of DHK or DHQ as a substrate. The amount of product of DeDFR1 and DeDFR2 was different. When using DHK as a substrate, the target product of DeDFR1 was comparable to that of DeDFR2. However, when using DHQ as a substrate, the target product of DeDFR2 was lower than DeDFR1 by approximately 24%. The above data provide evidence that the DeDFR1 and DeDFR2 in *D*. *erythrosora* had DFR activity. In addition, the substrate specificity of these enzymes differed from that of AtDFR, which could catalyze DHM.

### Site-directed mutagenesis of the substrate-specificity-determining site

**Fig 5:**
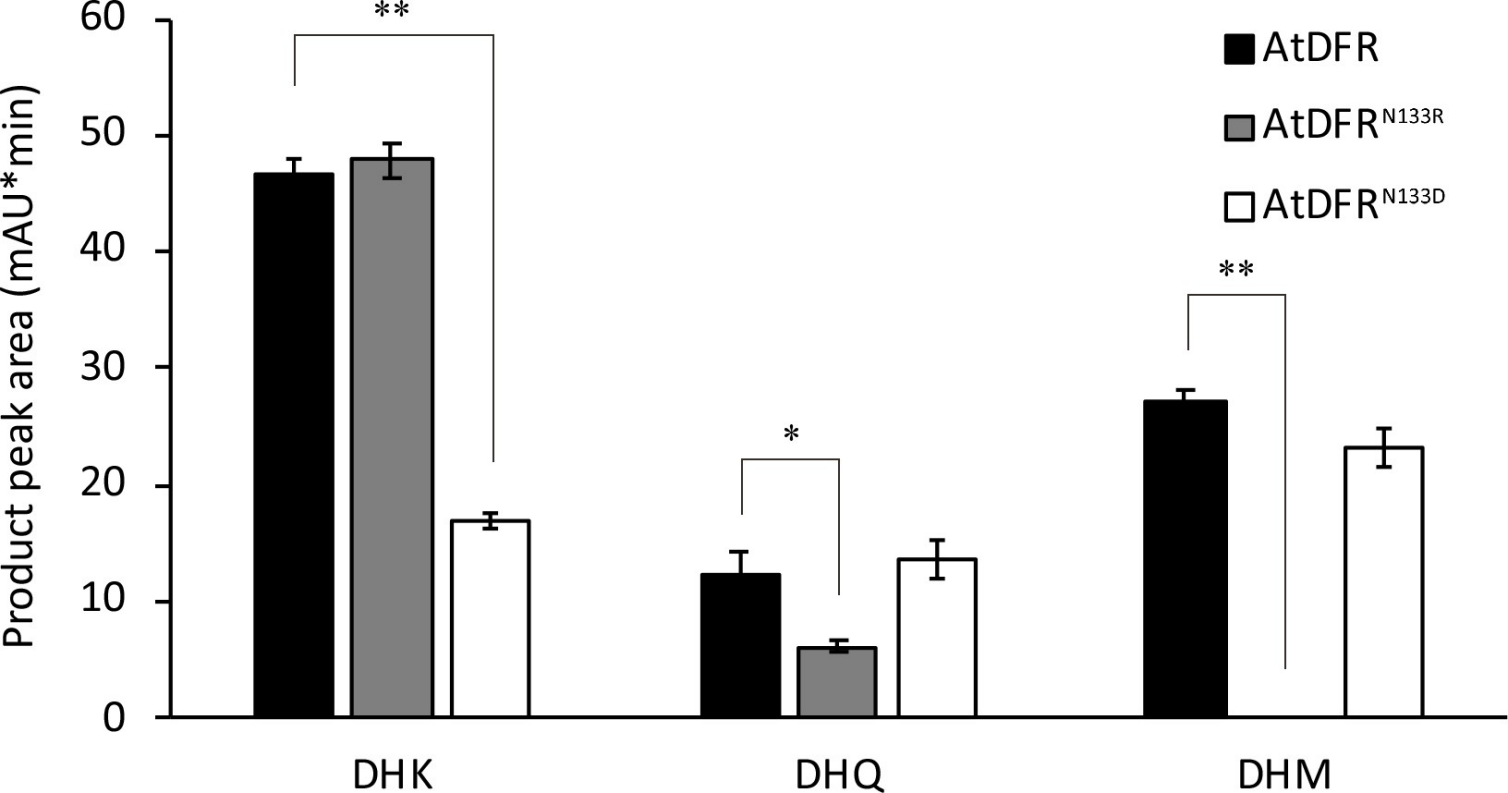
The product peak area of HPLC for modified AtDFR proteins with DHK, DHQ, and DHM as substrates. Bars represent the mean ± SD. *Statistically significant difference compared with AtDFR at 0.01<*p*<0.05. **Statistically significant difference compared with AtDFR at *p*<0.01.

We found that both DeDFR1 and DeDFR2 had an Arg at the substrate-specificity-determining site, and they did not have the same ability to catalyze DHM like the Asn-type DFR. To verify the substrate preferences of the Arg-type DFR, the overlap PCR method was used to modify AtDFR to AtDFR^N133D^ and AtDFR^N133R^ (S3 Fig). Because modification of the 133rd residue hardly affected the solubility of AtDFR, we analyzed the activity of the modified recombinant proteins by quantifying the total protein content. Crude extracts of *E*. *coli* expressing modified AtDFRs were subjected to enzyme assays using DHK, DHQ, and DHM as substrates in the presence of NADPH (S2 Fig). The results showed that the enzyme activity was changed after the site was modified (Fig 5). The catalytic capabilities of AtDFR, AtDFR^N133D^ and AtDFR^N133R^ were quite different. When DHK was used as a substrate, the AtDFR^N133D^ activities decreased significantly. However, when DHQ was used as a substrate, the target product of AtDFR^N133R^ yielded approximately half of that obtained with AtDFR. Notably, when DHM was used as a substrate, products were not detected with AtDFR^N133R^, as observed for DeDFR1 and DeDFR2. The site-directed mutagenesis results showed that the 133rd amino acid residue was indeed an important site affecting substrate specificity. They also showed that Arg-type DFRs could hardly catalyze DHM.

### Overexpression of *DeDFRs* in Arabidopsis *tt3-1* Mutant

**Fig 6:**
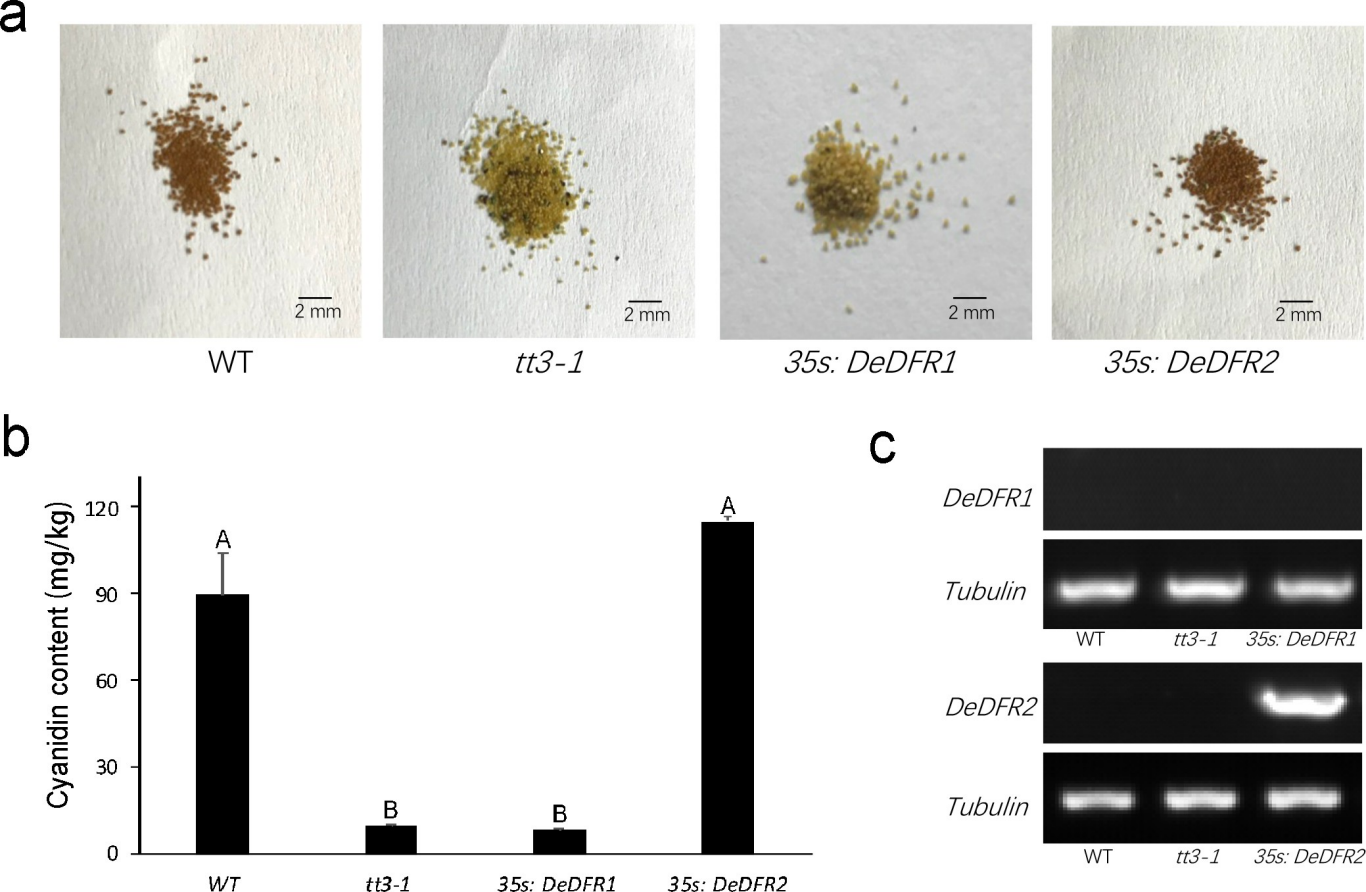
Overexpression of *DeDFR1* and *DeDFR2* in Arabidopsis *tt3-1* mutant. (a) Phenotype of the wild type (WT, Col), *tt3-1* mutant and transgenic lines seed coats. (b) HPLC analysis of cyanidin content in the seeds of wild-type, *tt3-1* mutant and transgenic lines. Three replicates were used for each sample. Bars represent the mean ± SD. Different capital letters above the bars indicate a significant difference between the samples at *p*<0.01. (c) Expressional analysis of *DeDFR1* and *DeDFR2* by RT-PCR in the leaves of wild-type, *tt3-1* mutant and transgenic lines.

The Arabidopsis *tt3-1* mutant is characterized by the knockout of *DFR*, resulting in a lack of anthocyanins, so the seed coat is yellow due to absence of pigment. To investigate the function of *DeDFRs* in plants, they were ectopically expressed in Arabidopsis *tt3-1* mutant. Using the flower dip transformation method, transgenic *DeDFR1* and *DeDFR2* plants were obtained (Fig 6A). The results showed that the *DeDFR1* gene could not be expressed in the transgenic plants, and the seeds of the transgenic plants were still yellow. By analyzing the codon preference, we found that unlike *AtDFR* or *DeDFR2*, multiple arginines were encoded by CGC in *DeDFR1*, which might have led to the lack of *DeDFR1* expression in Arabidopsis (S4 Fig). Phenotypic observation showed that transgenic plants expressing *DeDFR2* exhibited a restored brown color of the seed coat similar to wild type seeds. The anthocyanidin content (mainly cyanidin) in seeds of transgenic *DeDFR2* Arabidopsis was higher than in the mutant and wild type (Fig 6B). The presence and expression of the foreign gene in the transgenic lines were further confirmed by RT-PCR. The amplification of *DeDFR2* was observed in transgenic lines, not in wild type or mutants plants (Fig 6C). These results indicated that the overexpression of *DeDFR2* in the *tt3-1* mutant reconstructed the mutated anthocyanin biosynthesis.

## Discussion

The Arabidopsis *tt3-1* mutant is characterized by knockout of DFR, resulting in a lack of anthocyanins. Seed plant studies have shown that red/purple leaves or flowers are enriched in anthocyanins [38–41]. In *Malus crabapple*, the abundance of anthocyanins in red-leaf cultivars decreased with leaf maturation, but almost no anthocyanins were detected in the evergreen cultivar [42]. Similar to the above result, we found that the red young fronds had the highest anthocyanin content and that the greenish mature fronds had relatively low anthocyanin levels in *D*. *erythrosora*. Furthermore, researchers found that anthocyanin concentrations were positively correlated with *DFR* expression in a variety of plants, such as *B*. *oleracea*, *G*. *biloba* and *Pyrus communis* [16, 43, 44]. By analyzing the transcripts, we found that the anthocyanin content was highly consistent with the trend of changes in *DeDFR1* and *DeDFR2* expression. In *D*. *erythrosora*, *DeDFR1* and *DeDFR2* were initially expressed in rolled immature fronds to produce anthocyanins. The expression levels of *DeDFR1* and *DeDFR2* and total anthocyanins peaked in young fronds. After the fronds matured, the expression of *DeDFR1* and *DeDFR2* decreased, the anthocyanin content was diluted, and the color of the mature fronds gradually deceased in intensity. Similar phenomena also occur in *M*. *crabapple*; *McDFR1* is mainly expressed in the early stage of red leaf growth [42]. Thus, *DeDFR1* and *DeDFR2* may be associated with anthocyanin coloration in fronds of *D*. *erythrosora*.

Multiple *DFR* genes are present in plants. At least five different *DFRs* exist in *L*. *japonicus* [13]. The *P*. *hybrida* genome contains three *DFR* genes located on chromosomes 2, 4 and 6 [45]. Both *Medicago truncatul*a and *Fagopyrum esculentum* contain two different *DFRs* [12, 35]. The Arabidopsis genome contains only one *DFR* gene [19]. However, there have been few reports of *DFRs* in ferns, mainly due to the substantial differences between the genomes of ferns and seed plants, making it almost impossible to obtain functional genes by homologous cloning. Conversely, there is a lack of genomic data for ferns—only the genomes of *A*. *filiculoides* and *S*. *cucullata* have been reported [46]—so there are few molecular studies on ferns. Transcriptome sequencing technology provides a wealth of data for functional genetic studies of species without genomic data. In the present study, we found two *DFRs* from the transcriptomic data of *D*. *erythrosora*, five *DFRs* from the genomic data of *S*. *cucullata* and six *DFRs* from the genomic data of *A*. *filiculoides*. All of these genes belong to the SDR superfamily, with a conserved NAD(P)-binding domain and a substrate-binding domain. DFR evolution can be clearly understood by generating a phylogenic tree with a broad range of plant species. As shown in Fig 2, DFRs of ferns and seed plants were clearly classified into different branches. *DeDFR1*, *AfDFR1* and *ScDFR1* clustered into one branch, while *DeDFR2*, *AfDFR2*, *AfDFR3*, and *ScDFR2* clustered into another branch. Thus, it can be deduced that *DeDFR1* and *DeDFR2* are independently evolved and may have different functions in *D*. *erythrosora*.

Previous studies have indicated that amino acid residue 133 plays an important role in substrate specificity and classified DFRs into different types based on this site, i.e., Asn-type DFRs, Asp-type DFRs, and non-Asn/Asp-type DFRs, with the amino acid residue at the 133 position being asparagine (Asn), aspartic acid (Asp) and neither Asn nor Asp, respectively [37, 47]. Most previous reports have suggested that Asn-type DFRs and Asp-type DFRs have the function of catalyzing dihydroflavonol [13, 47, 48]. However, we did not find an Asn-type or Asp-type DFR in *D*. *erythrosora*, *S*. *cucullata* and *A*. *filiculoides*. Unlike seed plants, most of these DFR proteins in the three ferns have an arginine at the substrate-specificity-determining site. On the other hand, unlike Asn-type or Asp-type DFRs, DeDFR1 and DeDFR2 proteins cannot catalyze DHM. Thus, we speculated that there is a new DFR functional type in ferns, and we named it Arg-type DFR. The Arg-type DFR was not reported in the current research on seed plants, and it might be unique to ferns.

Site-directed mutagenesis is a good way to verify the contribution of a specialized amino acid residue to the activity and substrate preference of DFRs. Based on the experiments, we found that modification of the 133rd amino acid residue of AtDFR could change the enzymatic activity efficiency. The present investigation showed that the modified AtDFR^N133D^ exhibited a reduced ability to catalyze the conversion of DHK to leucopelargonidins, which is consistent with studies of *M*. *truncatula* and *L*. *japonicus* [12, 13]. Although Asp-type DFRs exhibit a certain catalytic ability toward DHK *in vitro*, pelargonidin-based anthocyanins are not detected in plants with Asp-type DFRs [26, 47]. We speculate that in plants with Asp-type DFRs, leucopelargonidins are converted to the highly stable cyanidin/delphinidin-based anthocyanins by F3’H or F3’5’H, making it difficult for the plants to produce pelargonidin-based anthocyanins. Conversely, we found that the modified AtDFR^N133R^ exhibited a reduced ability to catalyze DHQ, and we did not detect the product of DHM, which is similar to the catalytic effects observed for DeDFR1 and DeDFR2. This finding demonstrates that Arg-type DFRs are significantly different from the Asp and Asn types in terms of function. Notably, the product yields of DeDFR1 and DeDFR2 were significantly lower than AtDFR^N133R^. This finding indicates that in Arabidopsis, the structure of DFR is favorable for substrate binding, so the modified AtDFR^N133R^ is highly efficient. It is possible that other sites can affect binding efficiency via the spatial configuration.

In the present study, the 133rd amino acids of the functional DFRs were Arg, Asn and Asp, all of which are polar amino acids [35]. Studies on the crystal structure of DFRs from *V*. *vinifera* have shown that the side chains of N133 interact with both hydroxyl groups of ring B of DHQ. Moreover, the carboxyl and amino groups of N133 can make contacts with the ligand at C3' and C4’ of DHQ, respectively [25]. Based on the docking model [35], we analyzed the differences in binding among proteins with different 133rd amino acids and for different ligands. The Asp-type DFR is able to catalyze DHQ via the carboxyl group on the side chain of D133; however, it is difficult to catalyze DHK due to the lack of an amino group in contact with the hydroxyl group on C4’. The Arg-type DFR catalyzes DHQ inefficiently, mainly due to the lack of a carboxyl group on the side chain of R133. The Arg-type DFR can catalyze DHK as efficiently as the Asn-type DFR, mainly via the interaction of the amino group with the hydroxyl group on C4’. These findings explain why different types of DFRs have different catalytic capabilities for dihydroflavonols. In summary, the 133rd amino acid residue of DFRs is an important substrate recognition site, and different amino acid residues at this site directly affect the substrate binding ability.

**Fig 7:**
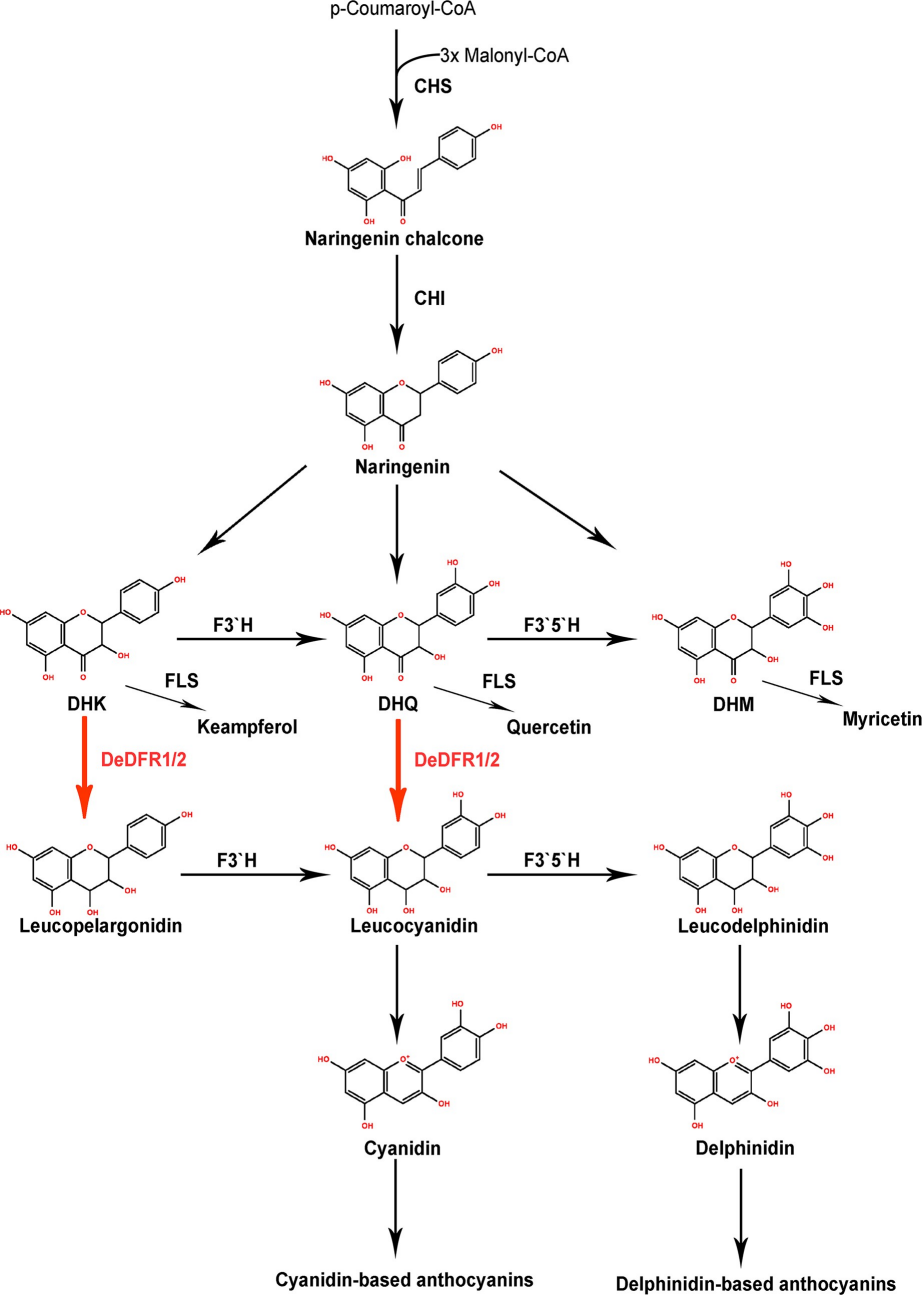
Proposed pathway for anthocyanin biosynthesis in *D*. *erythrosora*. The enzymes studied herein are represented in red font. Abbreviations: CHS, chalcone synthase; CHI, chalcone isomerase; DFR, dihydroflavonol 4-reductase; F3′H, flavonoid 3′-hydroxylase; F3′5′H, flavonoid 3′5′-hydroxylase; FLS, flavonol synthase; DHK, dihydrokaempferol; DHQ, dihydroquercetin; DHM, dihydromyricetin.

Catalytic activity analysis showed that the function of DeDFR1 and DeDFR2 is mainly to catalyze DHK and DHQ. Therefore, the expected products are pelargonidin-based and cyanidin-based anthocyanins, but we only detected cyanidin-based and delphinidin-based anthocyanins in D. *erythrosora*. The lack of pelargonidin-based anthocyanins might be due to the influence of downstream genes, such as F3’H and F3’5’H, which convert the product of DeDFR1 and DeDFR2 to a more stable structure with two or three hydroxyl groups on ring B (Fig 7). In previous studies, a few glycosylation compounds of quercetin, kaempferol, and myricetin were detected in *D*. *erythrosora* [29, 30], indicating that ferns have enzymes similar to FLS, which can compete with DFRs for substrates. Interestingly, Zhang et al. detected DHM in *D*. *erythrosora* [49], indicating that the functional enzymes acting on DHM are not sufficient for complete digestion of this substrate. This finding is consistent with the results showing that DeDFR cannot catalyze DHM. Whether the synthetic process of cyanidin-based and delphinidin-based anthocyanins is consistent with our hypothesis is worth exploring in the future. The development of plants is a synergistic process, and the synthesis of anthocyanins is not controlled by a single gene. The mechanism by which *DeDFR* and *F3’H/F3’5’H* synergistically control the anthocyanin type, the competition mechanism between *DeDFR* and *FLS*, and whether the process of downstream anthocyanin synthesis in ferns is the same as in seed plants remain to be further studied. The discovery of the new functional Arg-type DFR also provides a new direction for flower cultivation.

## Conclusions

The newly identified DeDFR1 and DeDFR2 genes were associated with the accumulation of anthocyanins in *D*. *erythrosora*. Moreover, both DeDFR1 and DeDFR2 enzyme proteins can catalyze DHK and DHQ but do not catalyze DHM. DeDFR1 and DeDFR2 are Arg-type DFRs, a previously unreported DFR type. This finding might provide new insights into the anthocyanin biosynthesis pathway in ferns.

## Supporting information

S1 Fig SDS-PAGE analysis of DFRs and catalytic activity of AtDFR. (a) Coomassie brilliant blue-stained polyacrylamide gel. M, protein marker. Lane 1, soluble lysate fraction from *E*. *coli* BL21 (DE3) harboring pET28a-AtDFR induced by IPTG. Lane 2, soluble lysate fraction from *E*. *coli* BL21 (DE3) harboring pET28a-DeDFR1 induced by IPTG. Lane 3, soluble lysate fraction from *E*. *coli* BL21 (DE3) harboring pET28a-DeDFR2 induced by IPTG. (b) DHK, DHQ, and DHM can be converted to pink compounds by AtDFR (1–3). DHK, DHQ, and DHM cannot be converted to colored compounds by the empty pET-28a (4–6). (c) Schematic HPLC chromatograms of the reaction products of the three substrates with AtDFR.
(TIF)

S2 Fig HPLC chromatograms of the reaction products of three substrates with DeDFRs and modified AtDFR. (TIF)

S3 Fig SDS-PAGE analysis of the site-directed mutagenesis of AtDFR protein. (a, b)The cDNA and deduced amino acid sequence of modified AtDFR. (c) Amplification of the *AtDFR*^*N133R*^ gene. M, DL2000 DNA marker. Lane 1, amplification of the 5 'end sequence. Lane 2, amplification of the 3 'end sequence. Lane 3, amplification of the full-length *AtDFR*^*N133R*^. (d) Amplification of the full-length modified AtDFR. M, DL2000 DNA marker. Lane 1, amplification of the full-length *AtDFR*. Lane 2, amplification of the full-length *AtDFR*^*N133R*^. Lane 3, amplification of the full-length *AtDFR*^*N133D*^. (e) Coomassie brilliant blue-stained polyacrylamide gel of modified AtDFR. M, protein marker. Lane 1, soluble lysate fraction from *E*. *coli* BL21 (DE3) harboring pET28a-AtDFR induced by IPTG. Lane 2, soluble lysate fraction from *E*. *coli* BL21 (DE3) harboring pET28a- *AtDFR*^*N133R*^ induced by IPTG. Lane 3, soluble lysate fraction from *E*. *coli* BL21 (DE3) harboring pET28a- *AtDFR*^*N133D*^ induced by IPTG.
(TIF)

S4 Fig Codon usage analysis of *AtDFR*, *DeDFR1* and *DeDFR2*. (TIF)

S1 Table List of primers used in this study. (DOCX)

S2 Table The theoretical molecular weight and isoelectric point for DFRs. (DOCX)

S1 Raw Images Original gel photos. (PDF)
