# Supplementary figures and images for: Arg-type dihydroflavonol 4-reductase genes from the fern Dryopteris erythrosora play important roles in the biosynthesis of anthocyanins
Source: PLoS One. 2020 May 1;15(5):e0232090. doi: 10.1371/journal.pone.0232090 (PMC7194404; doi:10.1371/journal.pone.0232090)

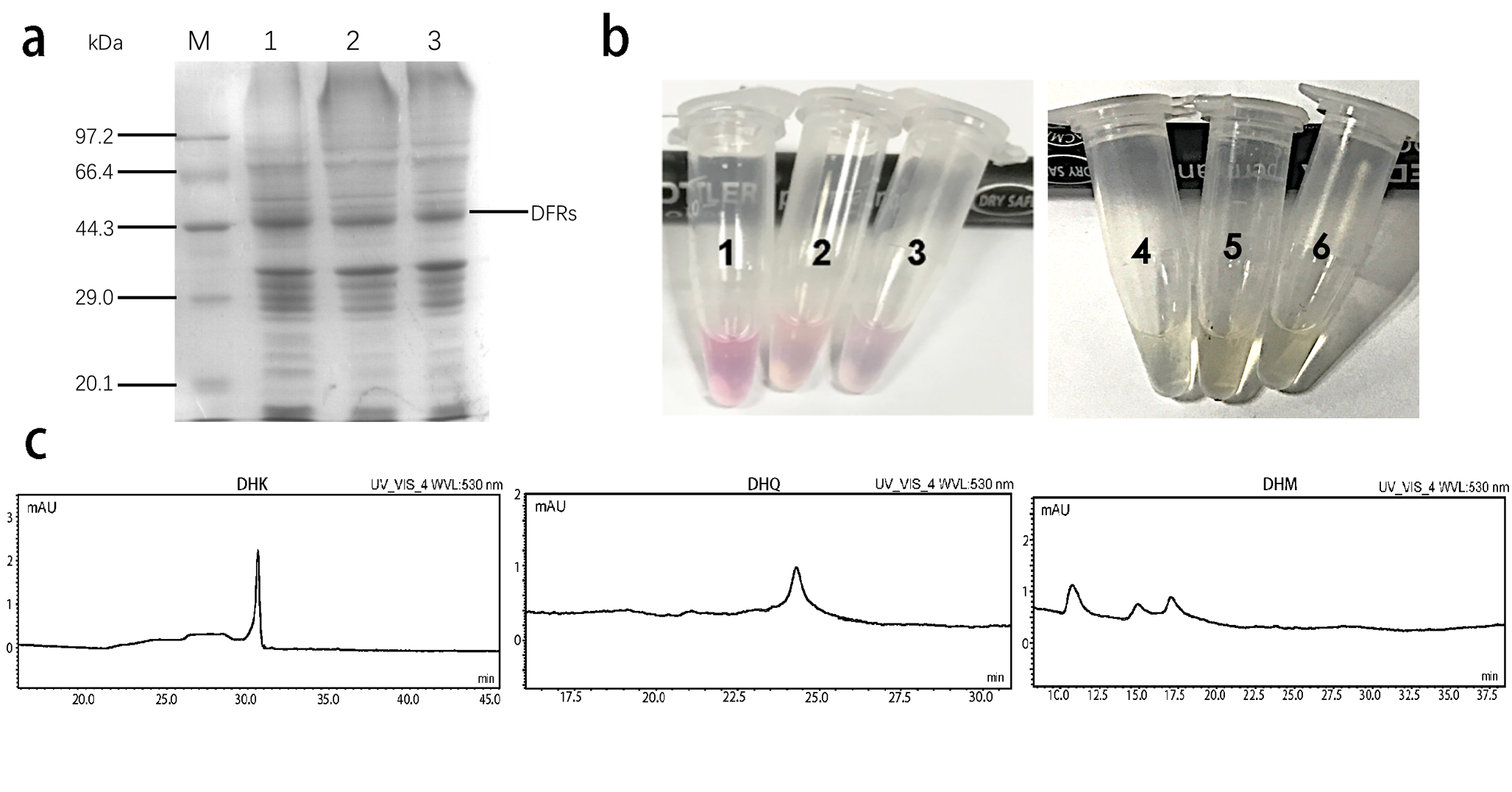

Supplement: S1 Fig — (a) Coomassie brilliant blue-stained polyacrylamide gel. M, protein marker. Lane 1, soluble lysate fraction from E. coli BL21 (DE3) harboring pET28a-AtDFR induced by IPTG. Lane 2, soluble lysate fraction from E. coli BL21 (DE3) harboring pET28a-DeDFR1 induced by IPTG. Lane 3, soluble lysate fraction from E. coli BL21 (DE3) harboring pET28a-DeDFR2 induced by IPTG. (b) DHK, DHQ, and DHM can be converted to pink compounds by AtDFR (1–3). DHK, DHQ, and DHM cannot be converted to colored compounds by the empty pET-28a (4–6). (c) Schematic HPLC chromatograms of the reaction products of the three substrates with AtDFR. (TIF) [file pone.0232090.s001.tif]

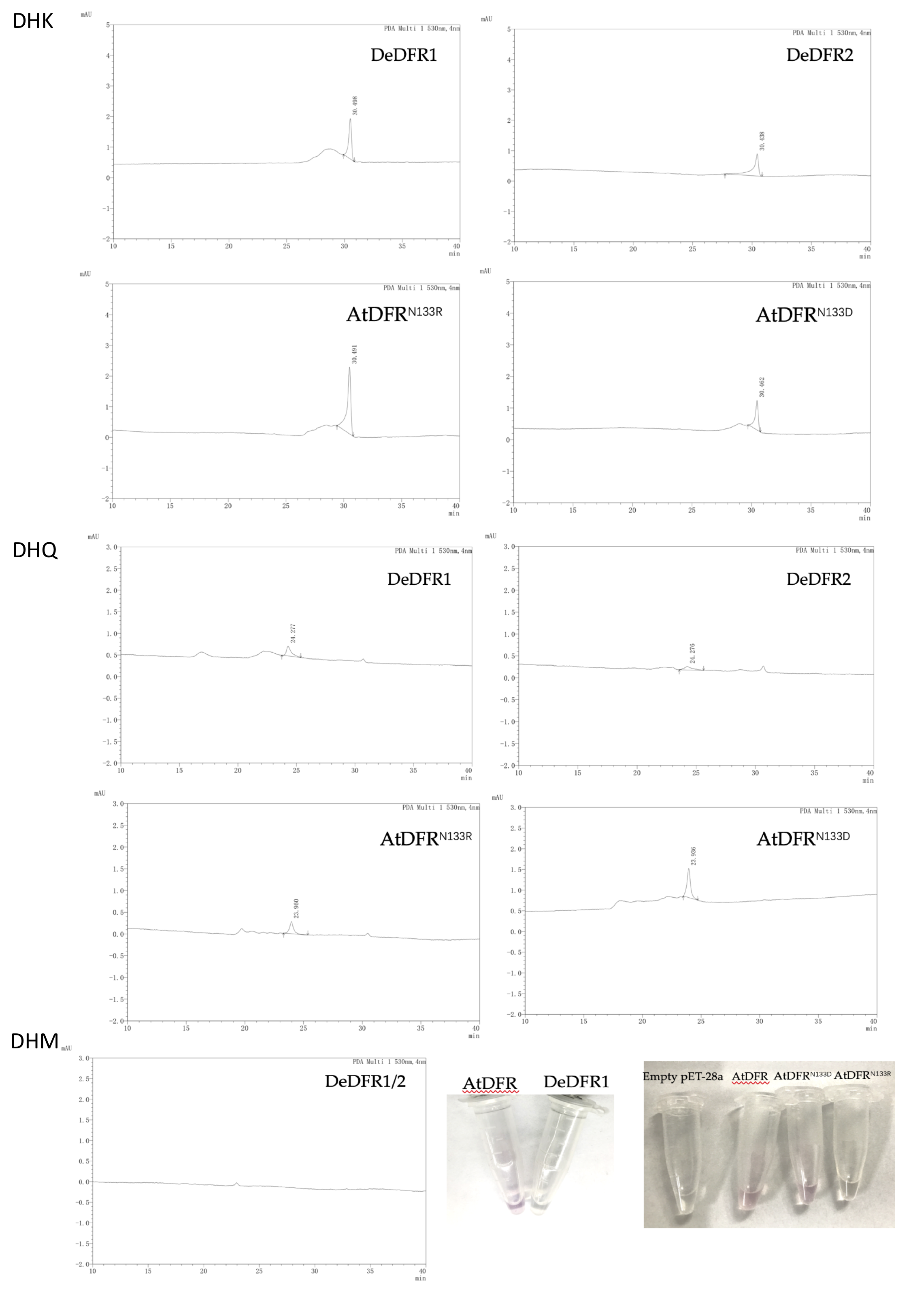

Supplement: S2 Fig — (TIF) [file pone.0232090.s002.tif]

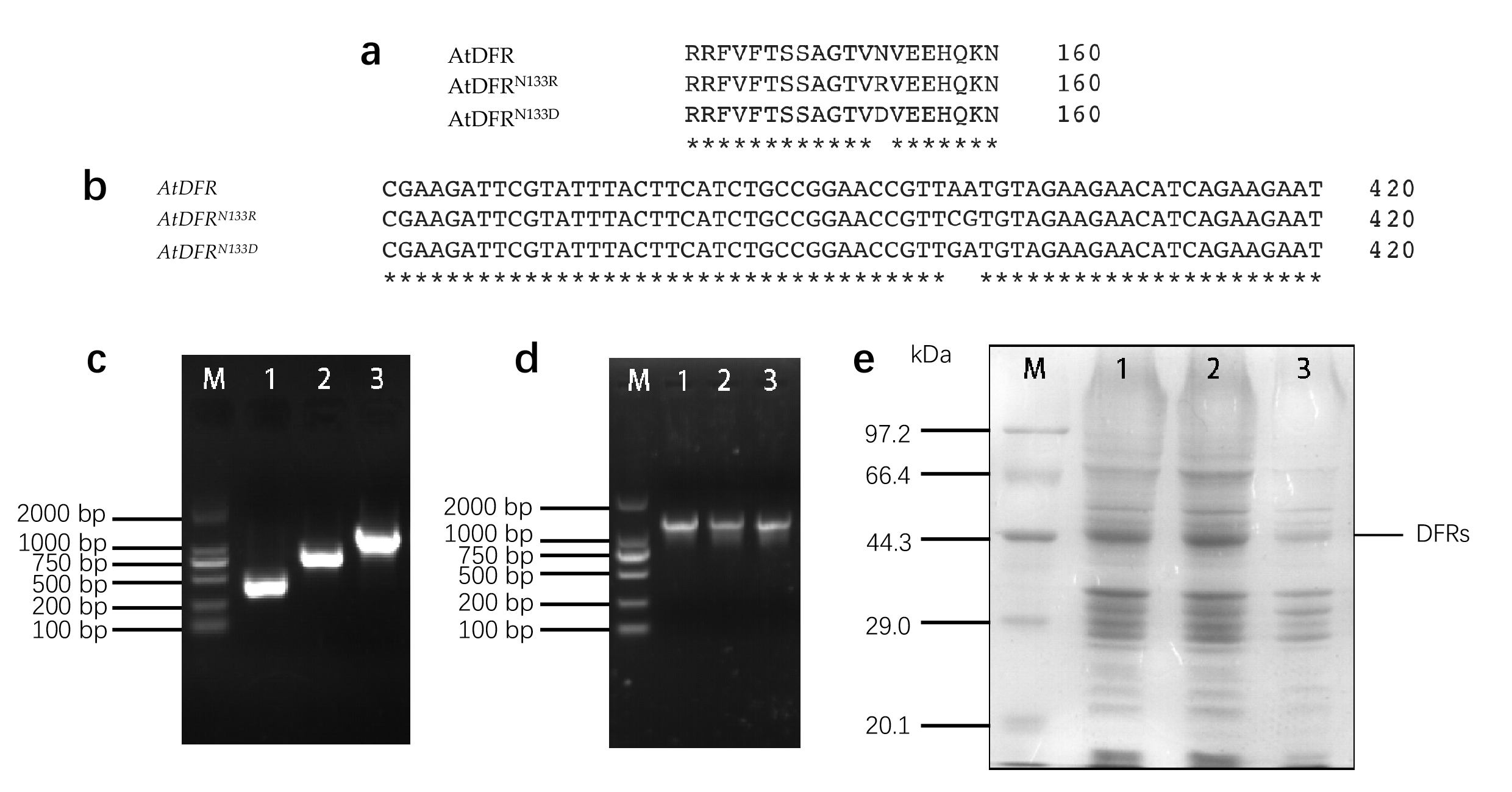

Supplement: S3 Fig — (a, b)The cDNA and deduced amino acid sequence of modified AtDFR. (c) Amplification of the AtDFRN133R gene. M, DL2000 DNA marker. Lane 1, amplification of the 5 'end sequence. Lane 2, amplification of the 3 'end sequence. Lane 3, amplification of the full-length AtDFRN133R. (d) Amplification of the full-length modified AtDFR. M, DL2000 DNA marker. Lane 1, amplification of the full-length AtDFR. Lane 2, amplification of the full-length AtDFRN133R. Lane 3, amplification of the full-length AtDFRN133D. (e) Coomassie brilliant blue-stained polyacrylamide gel of modified AtDFR. M, protein marker. Lane 1, soluble lysate fraction from E. coli BL21 (DE3) harboring pET28a-AtDFR induced by IPTG. Lane 2, soluble lysate fraction from E. coli BL21 (DE3) harboring pET28a- AtDFRN133R induced by IPTG. Lane 3, soluble lysate fraction from E. coli BL21 (DE3) harboring pET28a- AtDFRN133D induced by IPTG. (TIF) [file pone.0232090.s003.tif]

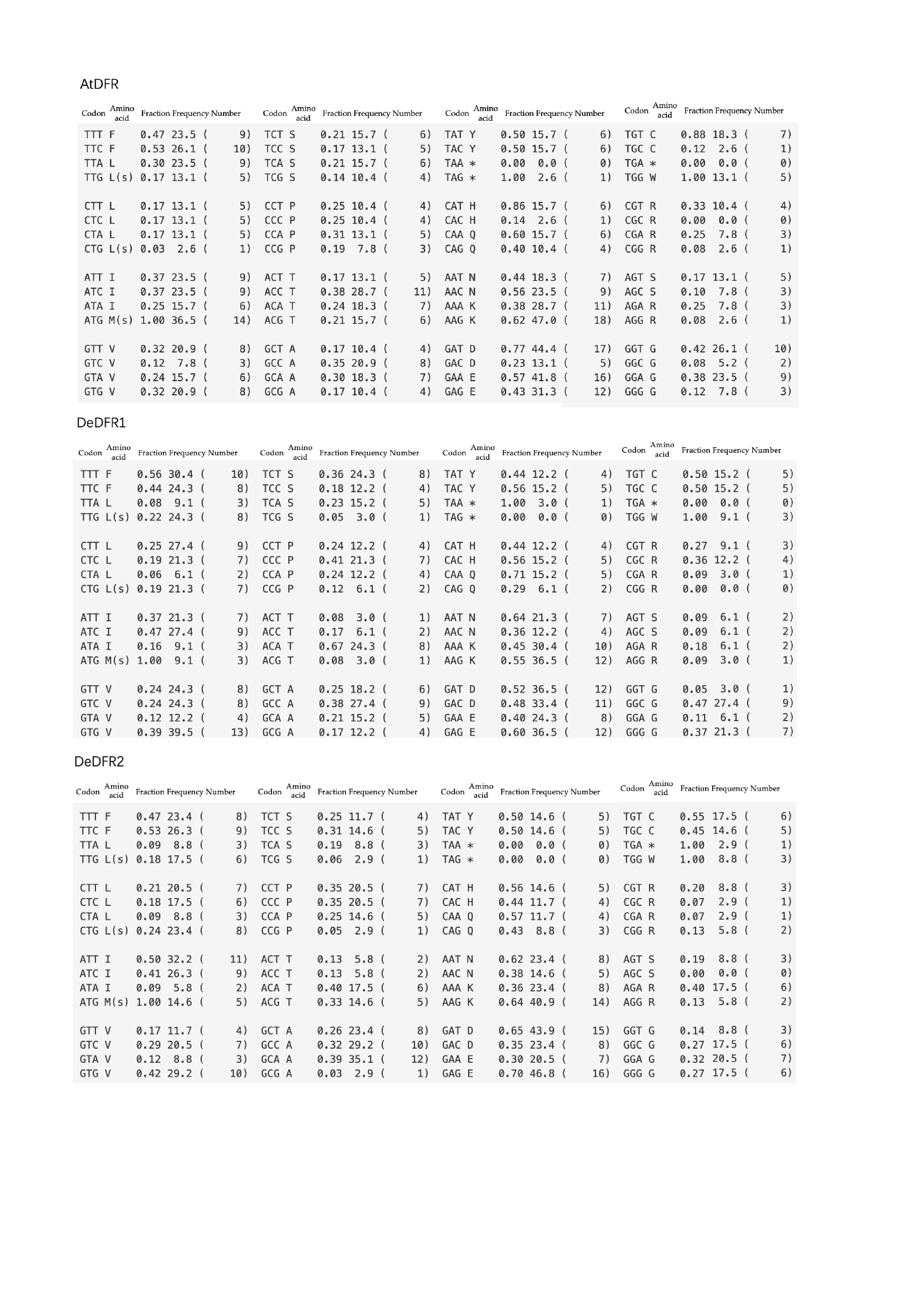

Supplement: S4 Fig — (TIF) [file pone.0232090.s004.tif]

Fig 6. c

DeDFR1

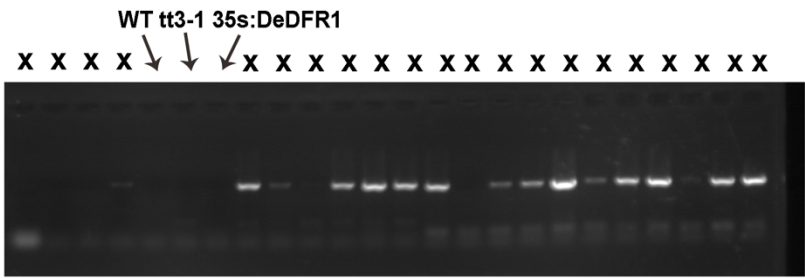

Tubulin

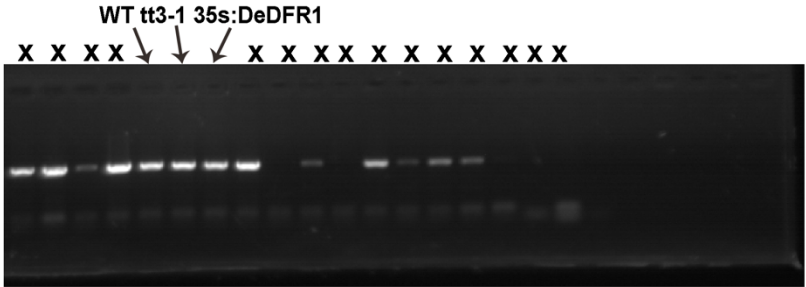

DeDFR2

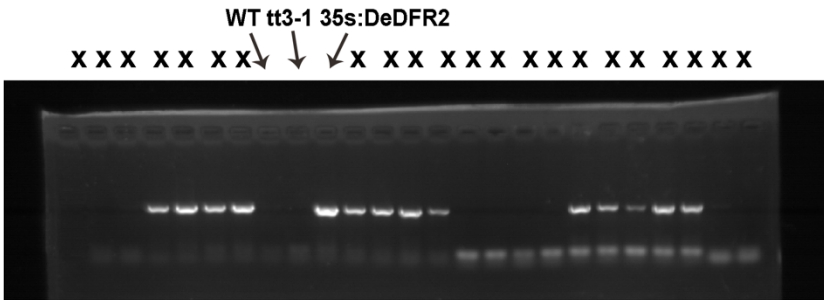

Tubulin

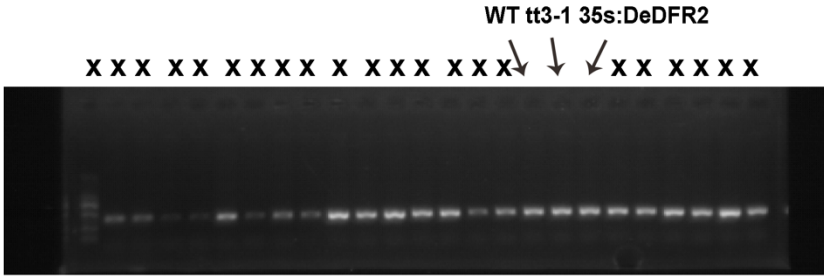

S1 Fig. a and S3 Fig. e

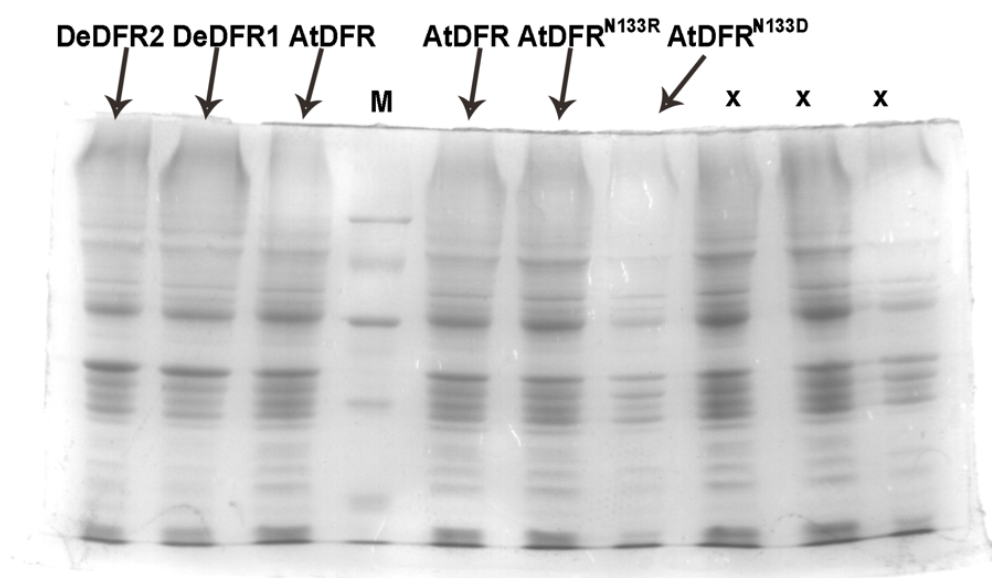

S3 Fig. c and d

c

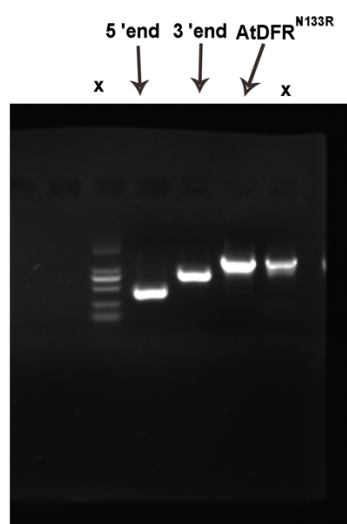

d

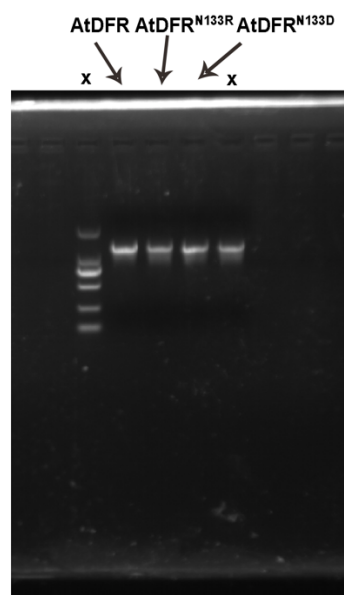

Supplement: S1 Raw Images — (PDF) [file pone.0232090.s007.pdf]
