# Supplementary material for: Arg-type dihydroflavonol 4-reductase genes from the fern Dryopteris erythrosora play important roles in the biosynthesis of anthocyanins
Source: PLoS One. 2020 May 1;15(5):e0232090. doi: 10.1371/journal.pone.0232090 (PMC7194404; doi:10.1371/journal.pone.0232090)
Supplement: S1 Table — (DOCX) [file pone.0232090.s005.docx]

**S1 Table. List of primers used in this study**

| **Primer name** | **Primer sequence (5'→3')** |
| --- | --- |
| *DeDFR1-28a-F* | aatgggtcgcggatcATGGACAAGCCCCTTCAC^a^ |
| *DeDFR1-28a-R* | caagcttgtcgacggTTATAACAGCCCTTTCTCCT |
| *DeDFR2-28a-F* | aatgggtcgcggatcATGGCTCCTAATGCTGTCG |
| *DeDFR2-28a-R* | caagcttgtcgacggTCAAGGGGCTTCTCCAG |
| *AtDFR-28a-F* | aatgggtcgcggatcATGGTTAGTCAGAAAGAGAC |
| *AtDFR-28a-R* | caagcttgtcgacggCTAGGCACACATCTGTTGTG |
| *DeGAPDH-qRT-F* | GTGGAGCTAAGAGAGTGGTGAT |
| *DeGAPDH-qRT-R* | CTGGGAATTATATTGAAGCCAG |
| *DeDFR1-qRT-F* | GAGAAAGCTGCGGTGGAGTT |
| *DeDFR1-qRT-R* | GCTGTTGGGAATGTTGGAAAG |
| *DeDFR2-qRT-F* | TCTCCCTTGTCACAGGCGAT |
| *DeDFR2-qRT-R* | CAGGACCCATTGTAACGACC |
| *AtDFR^N133R^-F* | GGAACCGTTCGTGTAGAAGAACA^b^ |
| *AtDFR^N133R^-R* | TGTTCTTCTACACGAACGGTTCC |
| *AtDFR^N133D^-F* | GGAACCGTTGATGTAGAAGAACA |
| *AtDFR^N133D^-R* | TGTTCTTCTACATCAACGGTTCC |
| *DeDFR1-pbi121-F* | cacgggggactctagATGGACAAGCCCCTTCAC |
| *DeDFR1-pbi121-R* | agggactgaccacccTAACAGCCCTTTCTCCTTG |
| *DeDFR2-pbi121-F* | cacgggggactctagATGGCTCCTAATGCTGTCG |
| *DeDFR2-pbi121-R* | agggactgaccacccAGGGGCTTCTCCAGGAT |
| *AtTUB-RT-F* | GGACACTACACTGAAGGTGCTGAG |
| *AtTUB-RT-R* | CAAGCTGATGAACAGAGAGAGTTG |
| *DeDFR1-RT-F* | GAGAAAGCTGCGGTGGAGTT |
| *DeDFR1-RT-R* | GCTGTTGGGAATGTTGGAAAG |
| *DeDFR2-RT-F* | AGAGAGGGTATAGAGTGCGTGC |
| *DeDFR2-RT-R* | CTCAAAGGAAAATTCAGTGG |

^a^The lowercase letters are vector linker sequences.

^b^The underline indicates the sequences modified by the point mutation.
