# Supplementary material for: Arg-type dihydroflavonol 4-reductase genes from the fern Dryopteris erythrosora play important roles in the biosynthesis of anthocyanins
Source: PLoS One. 2020 May 1;15(5):e0232090. doi: 10.1371/journal.pone.0232090 (PMC7194404; doi:10.1371/journal.pone.0232090)
Supplement: S2 Table — (DOCX) [file pone.0232090.s006.docx]

**S2 Table. The theoretical molecular weight and isoelectric point for DFRs**

| Given name | Theoretical pI | Theoretical Mw (Da) |
| --- | --- | --- |
| PhDFR | 5.97 | 42434.7 |
| NtDFR | 5.81 | 42437.6 |
| VvDFR | 5.89 | 37648.38 |
| AtDFR | 5.43 | 42774.96 |
| ZmDFR | 5.48 | 38783.51 |
| GbDFR | 5.27 | 38872.86 |
| DeDFR1 | 5.41 | 36337.94 |
| DeDFR2 | 5.54 | 37808.47 |
| AfDFR1 | 5.73 | 36381.82 |
| AfDFR2 | 5.65 | 39020.6 |
| AfDFR3 | 5.91 | 37938.58 |
| AfDFR4 | 6.41 | 36062.45 |
| AfDFR5 | 6.03 | 39314.28 |
| AfDFR6 | 6.57 | 36816.36 |
| ScDFR1 | 5.04 | 36072.46 |
| ScDFR2 | 5.65 | 38023.72 |
| ScDFR3 | 5.99 | 34989.05 |
| ScDFR4 | 6.38 | 36483.96 |
